# LINC00528 regulates myocardial infarction by targeting the miR-143-3p/COX-2 axis

**DOI:** 10.1080/21655979.2019.1704535

**Published:** 2019-12-27

**Authors:** Ketong Liu, Di Zhao, Di Wang

**Affiliations:** a Department of Cardiology III, The Third Affiliated Hospital of Qiqihar Medical University, Qiqihar, Heilongjiang, China; b Department of Cardiology I, The Third Affiliated Hospital of Qiqihar Medical University, Qiqihar, Heilongjiang, China; c Department of Gastroenterology I, The Third Affiliated Hospital of Qiqihar Medical University, Qiqihar, Heilongjiang, China

**Keywords:** Myocardial infarction, LINC00528, miR-143-3p, COX-2

## Abstract

This study is aimed to explore the roles of LINC00528 in myocardial infarction (MI) progression. Quantitative real-time PCR showed that the expression of LINC00528 and COX-2 was upregulated while miR-143-3p expression was down-regulated in post-MI cells. In function assays, LINC00528 suppression promoted post-MI cells proliferation and reduced cell apoptosis in vitro. In mechanism, LINC00528 interacted with miR-143-3p in post-MI cells. COX-2 served as a target of miR-143-3p in post-MI cells. Besides, LINC00528 inhibition on COX-2 expression and post-MI cells progression could be partially abolished by miR-143-3p inhibitors. Therefore, our findings suggested that LINC00528 exerted its regulatory roles in MI via the miR-143-3p/COX-2 axis, which provided a potential therapeutic target for MI patients treatment.

## Graphical Abstract

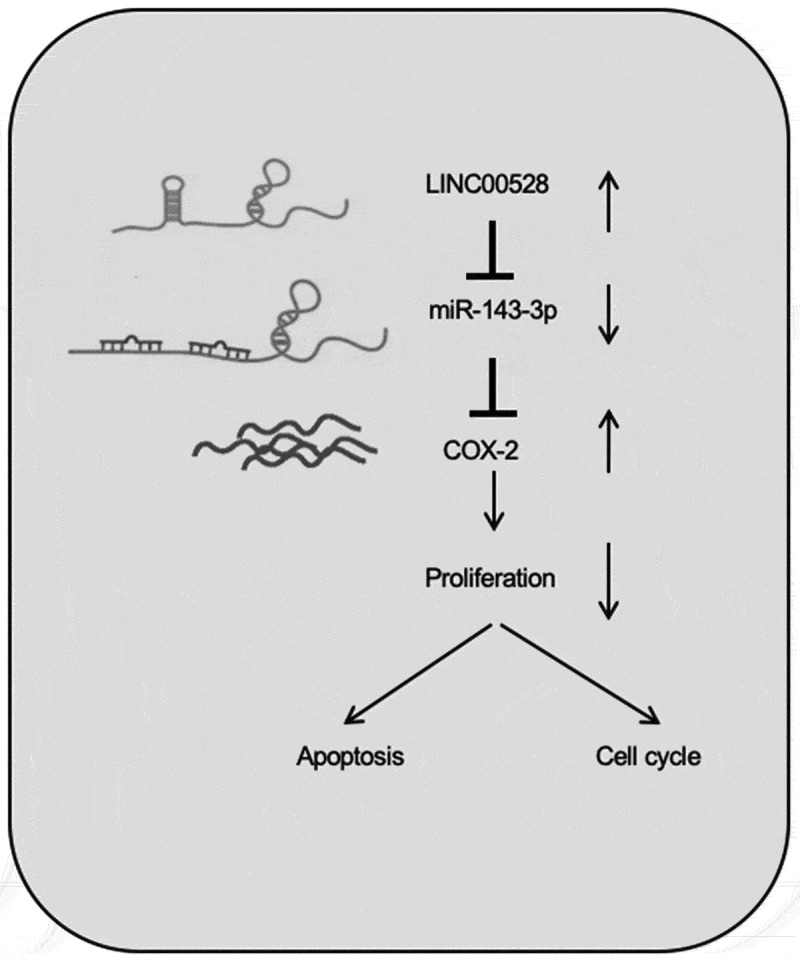

## Introduction

Myocardial infarction (MI) is the most serious cardiovascular disease (CVD) with high morbidity and mortality in the worldwide [1,2]. MI is characterized by myocardial necrosis caused by acute or persistent myocardial ischemia or hypoxia [3]. Despite great advancements in the treatment of MI over the past few decades, the prognosis for patients with MI remains unsatisfactory [4].

Long non-coding RNAs (lncRNAs) are a kind of non-coding RNAs that transcripts longer than 200 nucleotides, with no protein-coding function [5,6]. LncRNAs play important roles in many processes of cellular functions, for instance, X chromosome imprinting, chromatin modification, and immune response [7,8]. Recently, emerging evidence showed that lncRNAs have a high degree of tissue specificity in myocardial tissues, and play critical roles in remodeling and regeneration of cardiac myocytes [9]. For example, Micheletti et al showed that lncRNA Wisper could reduce the pathological development of cardiac fibrosis in response to MI and prevent adverse remodeling in the damaged heart [10]. Cai et al found that lncRNA CAREL regulated cardiomyocyte proliferation and heart regeneration in postnatal and adult heart after injury by miR-296/Trp53inp1/Itm2a axis [11]. Recently, Hu et al reported that lncRNA MALAT1 inhibition attenuated acute MI through miR-320-Pten axis [12]. Wang et al showed that lncRNA APF regulated autophagy and myocardial infarction through miR-188-3p/ATG7 axis [13]. However, the roles and underlying mechanism of lncRNAs in MI are still largely unclear.

In the present study, we found that LINC00528 and COX-2 expression were up-regulated while miR-143-3p expression was down-regulated in MI cells. Function assays revealed that LINC00528 overexpression reduced myocardial cells proliferation and increased cell apoptosis in vitro. In mechanism, LINC00528 acted as a ceRNA for miR-143-3p modulate COX-2 expression in MI. The LINC00528 might be an effective target for MI treatment.

## Materials and method

### Cell culture and transfection

The myocardial cell line H9c2 was purchased from BeNa Culture Collection (Beijing, China). The cell was maintained in Dulbecco’s modified eagle medium (DMEM) medium containing 10% fetal bovine serum (FBS, Gibco, Grand Island, NY, USA) and 100 μg/mL penicillin-streptomycin at 37ºC and 5% CO_2_.

Overexpression plasmids for LINC00528 (pcDNA3.1-LINC00528) were purchased from Shanghai GenePharma (Shanghai, China). miR-143-3p mimics, miR-143-3p inhibitor and their negative controls were purchased from RiboBio (Guangzhou, China). After cultured to 70–80% confluence, cells were transfected with pcDNA3.1-LINC00528, or miR-143-3p mimics, miR-143-3p inhibitors at a concentration of 50 nmol/l using Lipofectamine 3000 (Invitrogen, Carlsbad, CA, USA) according to the manufacturer’s instruction.

### Induction of myocardial infarction (MI)

Induction of myocardial infarction was according to previous studies [12,13].

### Real-time quantitative PCR

RNA was extracted from cells with TRIzol reagent (Invitrogen, Carlsbad, CA, USA). After RNA concentration and purity were examined, cDNA was synthesized using Reverse Transcription Kit (Takara, Dalian, China). With cDNA as the template, the expression levels of LINC00528, miR-143-3p and COX-2 mRNA were detected according to the instructions of the SYBR Green Premix Ex Taq II (Takara, Dalian, China). The reaction conditions were as follows: denaturation at 95ºC for 15s, annealing at 55ºC for 30s and extension at 72ºC for 10s. A total of 30 cycles of amplification were performed. Relative expression quantity was calculated by the 2^−△△Ct^ method.

### CCK-8 assay

Cell Counting Kit-8 (CCK-8, Promega, Shanghai, China) was used to detect cell proliferation ability. Transfected cells (3 × 10^3^/well) were planted in 96-well plates. After cells were cultured for 24 h, 48 h, 72 h, and 96 h, each well was added by 2 0μl of CCK-8 and incubated at 37°C for 2 h. The cell viability was calculated at a wavelength of 450nm using the FLx800 fluorescence microplate reader (BioTek, Winooski, VT, USA).

### Cell apoptosis assay

Transfected cells in 6-well plates were collected for washing in phosphate buffered saline (PBS). After double-staining with Annexin V fluorescein isothiocyanate (FITC)/propidium iodide (PI) detection kit (Sigma-Aldrich, St. Louis, MO, USA) for 15 min, apoptosis cells were assayed by flow cytometer (Beckman Coulter, Brea, CA, USA).

### Dual-luciferase reporter

The LINC00528 cDNA with miR-143-3p mutant binding sites and LINC00528 cDNA with miR-143-3p binding sites were synthesized and inserted into luciferase reporter vector pGL3 (Promega, Fitchburg, WI, USA), and titled as Mut-LINC00528 and Wt-LINC00528, respectively. The reporter plasmid and miR-143-3p mimics were co-transfected into HEK293T cells by Lipofectamine 2000. Luciferase activity was evaluated utilizing Dual-Luciferase Reporter Assay System (Promega).

### Western blot analysis

Cells were collected and subjected to total protein extractions using RIPA solution. Following denaturing at 95°C for 5 min, protein samples were subjected to 12% SDS-PAGE gel electrophoresis. Following gel transfer to PVDF membranes, blocking was performed in 5% nonfat milk for 2h at room temperature following by incubation with primary antibodies at 4°C overnight. Then, membranes were further incubated with secondary antibody for 2 h at room temperature. Finally, immunoactive blots were assayed by enhanced chemiluminescence solution (Millipore, Billerica, MA, USA).

### Statistical analysis

The statistical analysis was performed by using SPSS 20.0 software (Chicago, IL, USA). Data showed as mean ± SD from at least three separate experiments. Student’s t-test or One-way ANOVA was used to analyze the differences between different groups. *P*< 0.05 was considered statistically significant.

## Results

### LINC00528 expression in MI

**Figure 1.**
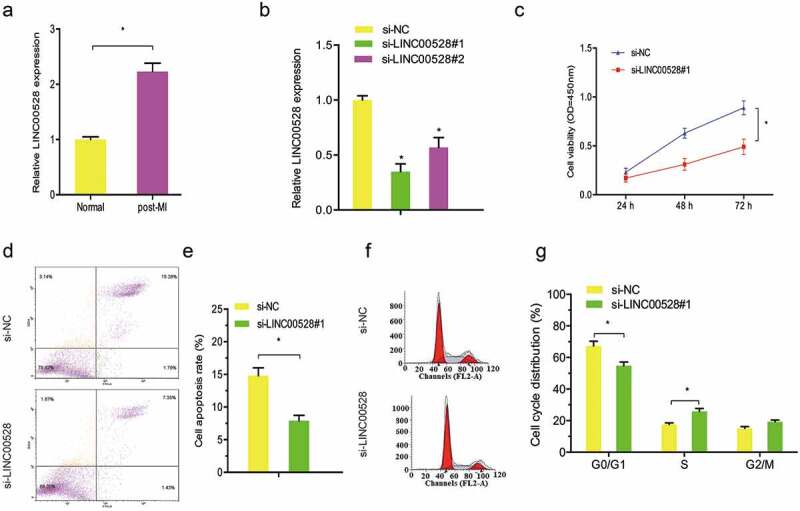
LINC00528 suppression increased post-MI cells viability. (a) LINC00528 was significantly increased in post-MI cells compared with normal cells. (b) qRT-PCR was used to determine the knockdown efficiency of si-LINC00528 on post-MI cells. (c) LINC00528 inhibition promoted post-MI cells proliferation. (d, e) LINC00528 inhibition reduced post-MI cells apoptosis. (f, g) LINC00528 inhibition promoted post-MI cells from G0/G1 into S and G2/M phase. *P < 0.05.

LncRNAs have been highlighted as key factors in the progression of AMI. Recently, studies showed that LINC00528 was one of the most upregulated lncRNA in coronary heart disease [14,15]. However, the roles and underlying mechanisms of LINC00528 in MI remain unclear. In the present study, qRT-PCR showed that LINC00528 was significantly increased in post-MI cells compared with normal cells (Figure 1(a)). Subsequently, we decreased LINC00528 expression in post-MI cells (Figure 1(b)). CCK-8 assay showed that LINC00528 inhibition significantly promoted post-MI cells proliferation compared to si-NC group (Figure 1(c)). Flow cytometry assay showed that LINC00528 inhibition reduced post-MI cells apoptosis and promoted post-MI cells from G0/G1 into S and G2/M phase (Figure 1(d–g)).

### Relationship between LINC00528 and miR-143-3p in myocardial cells

**Figure 2.**
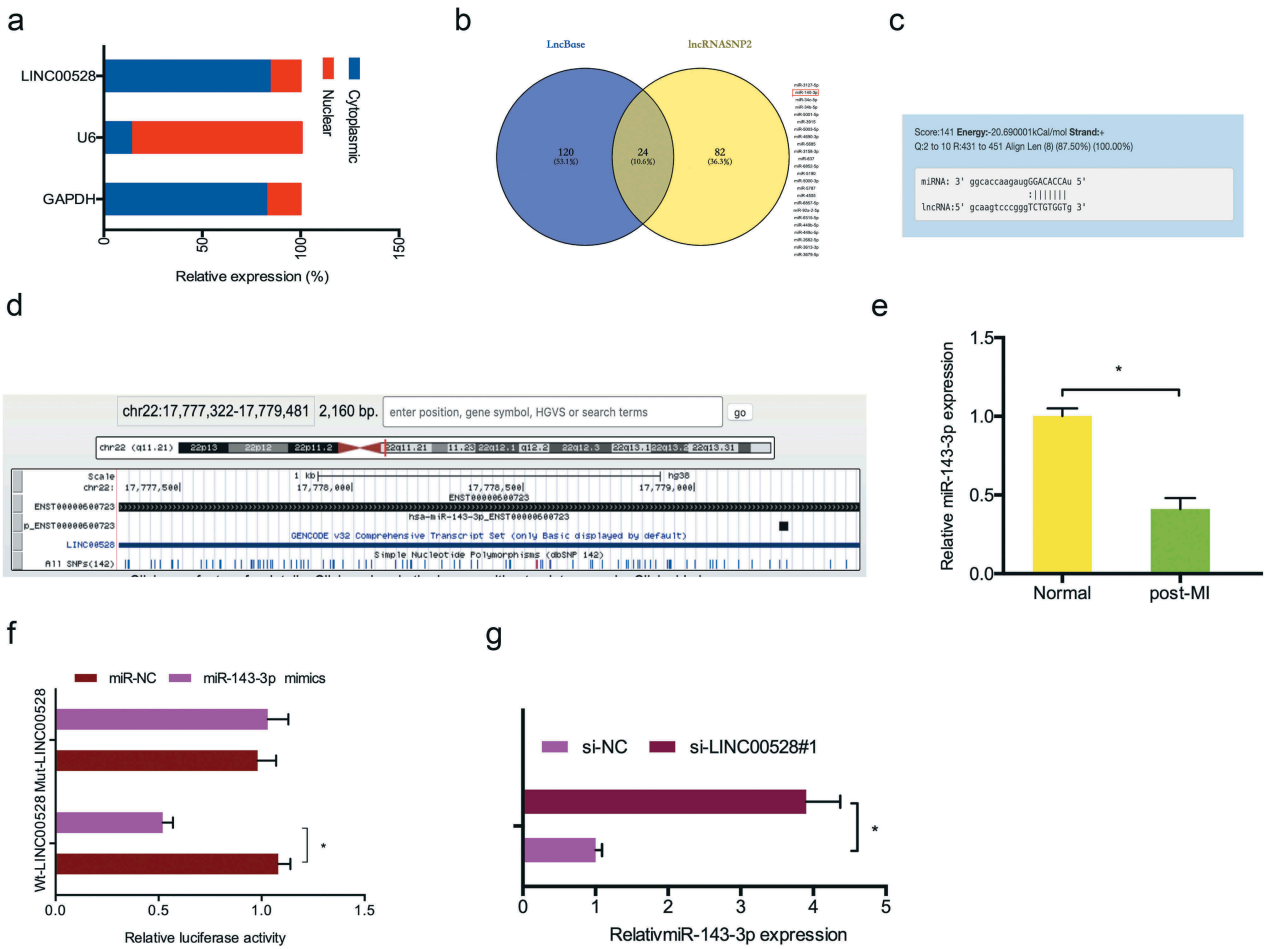
LINC00528 acted as a sponge of miR-143-3p. (a) Comparison of the abundance of LINC00528 in nuclear and cytoplasmic. (b–d) The predicting binding site of miR-143-3p and LINC00528. (e) MiR-143-3p expression was decreased in post-MI cells. (f) MiR-143-3p mimics reduced luciferase activities of LINC00528-Wt. (g) LINC00528 knockdown increased the expression of miR-143-3p in post-MI cells. *P < 0.05.

To explore the underlying mechanism of LINC00528 in MI, we firstly determined the location of LINC00528 in post-MI cells. Nuclear-cytoplasmic fractionation assay showed that LINC00528 was mainly localized in cytoplasm (Figure 2(a)). Subsequently, we analyzed the potential targets of LINC00528 via bioinformatics method (LncBase and lncRNASNP2), and miR-143-3p ranked top among those candidates (Figure 2(b–d)). Afterward, qRT-PCR showed that miR-143-3p expression was significantly decreased in post-MI cells compared with normal cells (Figure 2(e)). Luciferase reporter assay showed that miR-143-3p mimics significantly reduced the luciferase activities of LINC00528-WT group (Figure 2(f)). In addition, LINC00528 knockdown increased miR-143-3p expression in post-MI cells (Figure 2(g)). These data suggested that LINC00528 might serve as a sponge of miR-143-3p in myocardial cells

### Relationship between miR-143-3p and COX-2 in myocardial cells

**Figure 3.**
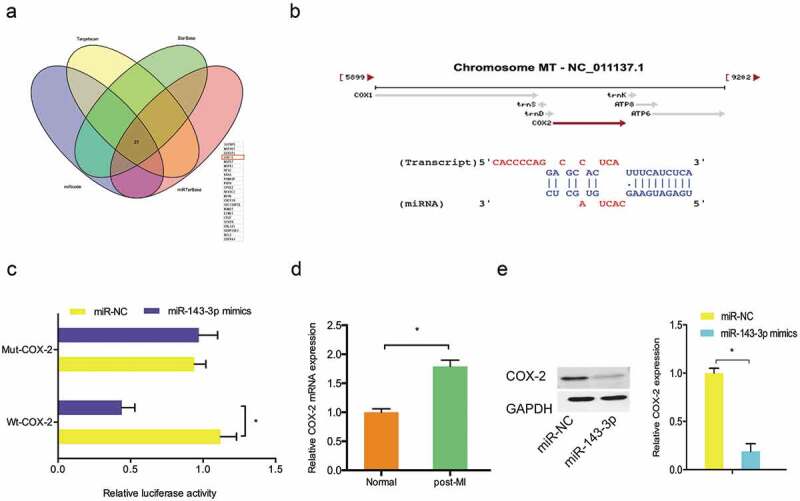
COX-2 acted as a target of miR-143-3p. (a, b) COX-2 was a potential putative target gene of miR-143-3p. (c) MiR-143-3p mimics inhibited luciferase activities of LINC00528-Wt group. (d) COX-2 was upregulated in post-MI cells. (e) MiR-143-3p mimics decreased COX-2 protein expression in post-MI cells. *P < 0.05.

Then, we identified the target of miR-143-3p. Results showed COX-2 was one of the most putative target genes of miR-143-3p (Figure 3(a,b)). Luciferase reporter assay showed that miR-143-3p mimics attenuated the luciferase activity of COX-2-Wt rather than COX-2-Mut (Figure 3(c)). In addition, qRT-PCR showed that the expression of COX-2 was significantly upregulated in post-MI cells compared to normal cells (Figure 3(d)). Western blot showed that miR-143-3p mimics decreased COX-2 protein expression in post-MI cells (Figure 3(e)). These data indicated that COX-2 might serve as a target of miR-143-3p in MI.

### Linc00528/143-3p/cox-2 axis in myocardial cells

**Figure 4.**
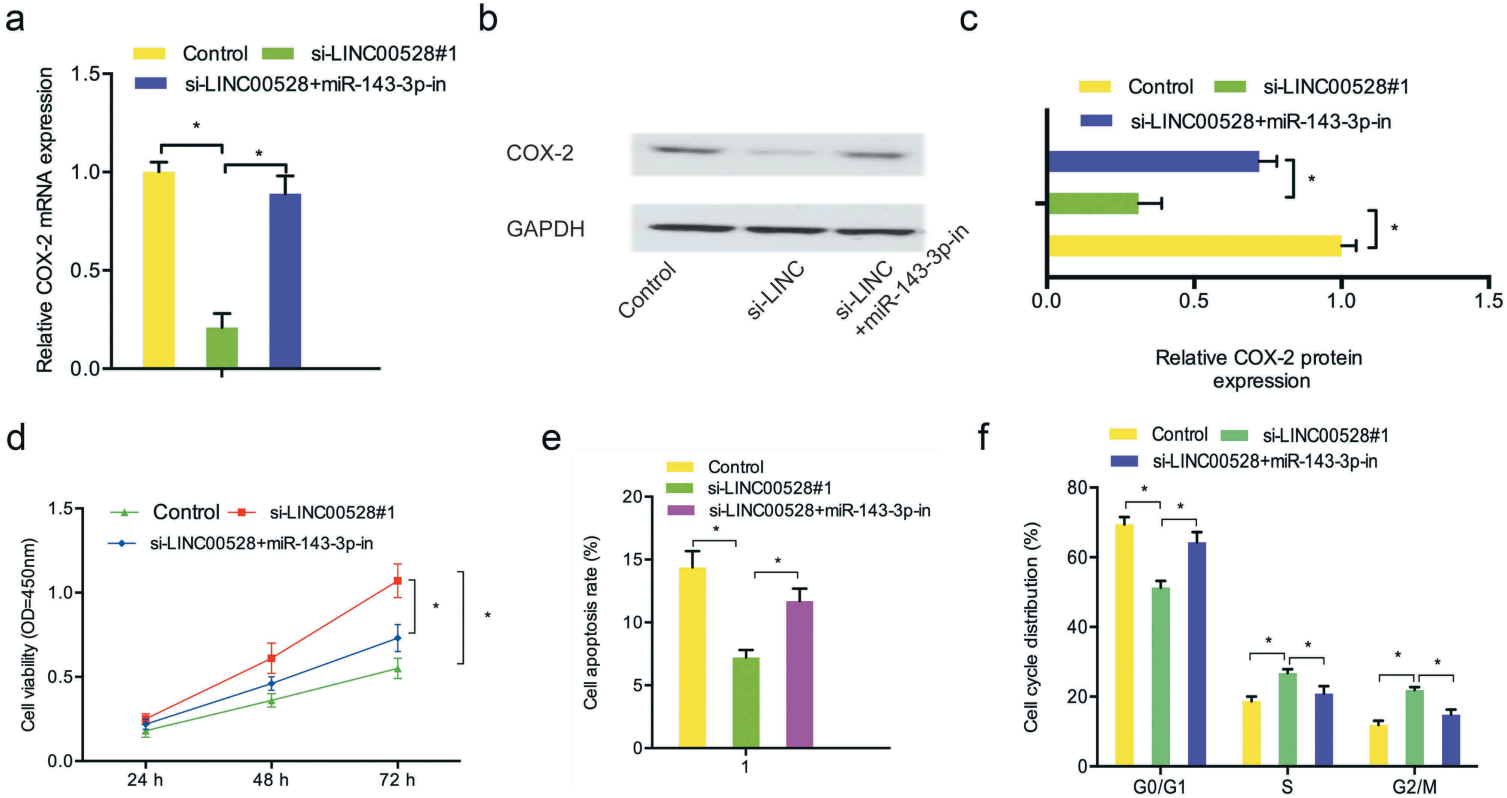
LINC00528/miR-143-3p/COX-2 axis in MI. (a–) MiR-143-3p inhibitors rescued the effects of LINC00528 inhibition on COX-2 expression. (d) MiR-143-3p inhibitors abolished the effects of si-LINC00528 on post-MI cells proliferation. (e, f) LINC00528 inhibition on post-MI cells apoptosis and cell cycle could be rescued by miR-143-3p inhibitors. *P < 0.05.

To further explore whether LINC00528 exerted its roles in myocardial cells by miR-143-3p/COX-2 axis. We firstly explored the expression of COX-2 in post-MI cells transfected with si-LINC00528 and miR-143-3p inhibitors. Results showed that LINC00528 downregulation significantly decreased COX-2 expression in post-MI cells, while miR-143-3p inhibitors rescued the effects (Figure 4(a–c)). Next, rescue assays were used to further confirm the axis. CCK-8 assay showed that miR-143-3p inhibitors abolished the effects of LINC00528 inhibition on post-MI cells proliferation (Figure 4(d)). Flow cytometry assays revealed that the effects of LINC00528 inhibition on post-MI cells apoptosis and cell cycle could be rescued by miR-143-3p inhibitors (Figure 4(e–f)). These results suggested that LINC00528 could regulate MI progression by miR-143-3p/COX-2 axis.

## Discussion

Recently, plenty of studies showed lncRNAs play critical roles in MI progression. For example, Li et al showed that lncRNA KCNQ1OT1 suppression protected against myocardial ischemia following acute MI [16]. Liu et al showed that lncRNA CAIF suppressed autophagy and attenuated MI through blocking p53-mediated myocardin transcription [17]. Zhang et al showed that lncRNA GAS5 regulated MI through regulating miR-525-5p/CALM2 axis [18]. However, the roles and underlying mechanism of lncRNA in MI remain unclear.

Recently, Guo et al showed that LINC00528 was one of the most upregulated lncRNA in MI tissues compared to normal tissues [15]. Zhou et al revealed that LINC00528 was one of the most upregulated lncRNA in CHD tissues compared with normal tissues [14]. However, the roles and underlying mechanisms of LINC00528 in MI progression remain unclear. In the present study, we confirmed that LINC00528 was upregulated in post-MI cells compared with normal cells. Furthermore, CCK-8 assay showed that LINC00528 suppression promoted post-MI cells proliferation. Flow cytometry assay revealed that LINC00528 inhibition decreased post-MI cells apoptosis and promoted post-MI cells from G0/G1 into S and G2/M phase. These data suggested that LINC00528 might play critical roles in MI progression.

Recently, dysregulation of miRNAs was reported in the development and progression of MI [19,20]. For example, Bayoumi et al showed that miR-532 could protect the heart in acute MI [21]. Chun et al found that miR-130 inhibition alleviated infarction-induced MI by promoting PPAR-γ expression [22]. Yu et al found that miR-143-3p could attenuate myocardial hypertrophy by reducing inflammatory response [23]. Moreover, the miRNA-lncRNA interaction in ischemia heart disease became an interest topic [24,25]. In the current study, we showed LINC00528 was mainly located in cytoplasm, suggesting that LINC00528 might regulate MI progression through ceRNA network. Bioinformatic analysis revealed that LINC00528 has a binding site of miR-143-3p. Subsequently, we confirmed the interaction of miR-143-3p and LINC00528 in post-MI cells by qRT-PCR and dual-luciferase reporter assays. Thus, these data indicated that LINC00528 might exert its biological function through as a sponge of miR-143-3p.

Recently, increasing studies showed that COX-2 was involved in MI progression. For example, Abbate et al showed that COX-2 was highly expressed in cardiomyocytes in patients with MI and positively associated with cells apoptosis [26]. Saito et al showed that COX-2 was highly expressed in MI zone, and COX-2 inhibitor reversed the effects [27]. In the present study, COX-2 was identified as a downstream gene of miR-143-3p. The expression of COX-2 was reduced by LINC00528 suppression in post-MI cells and miR-143-3p inhibitors abolished the effects. In addition, functional rescue assays suggested that miR-143-3p inhibitors rescued the effects caused by LINC00528 suppression on post-MI cells proliferation. Thus, we suggested that LINC00528 might regulate MI progression via the miR-143-3p/COX-2 axis.

## Conclusions

Our study manifested that LINC00528 contributed to MI progression through acting as a ceRNA of miR-143-3p to regulate the expression of COX-2, which might shed new light on the discovery of therapeutic strategies for MI patients.

## Highlights

- LINC00528 was upregulated in post-MI.
- LINC00528 suppression promoted myocardial cells proliferation.
- LINC00528 acted as sponge of miR-143-3p in myocardial cells.
- COX-2 acted as a target of miR-143-3p in myocardial cells.
